# Supplementary material for: Phylogenetic rewiring in mycorrhizal–plant interaction networks increases community stability in naturally fragmented landscapes
Source: Commun Biol. 2019 Dec 5;2:452. doi: 10.1038/s42003-019-0700-3 (PMC6895200; doi:10.1038/s42003-019-0700-3)

Supplementary Materials for:

Phylogenetic rewiring in mycorrhizal-plant interaction networks increases community stability in fragmented landscapes

Supplementary Figure 1.

Map showing the location and elevation of the 15 fragments used in this study. The numbers identify each fragment, and correspond to their size order. Details of the characteristics of each fragment are described in Supplementary Table 3.

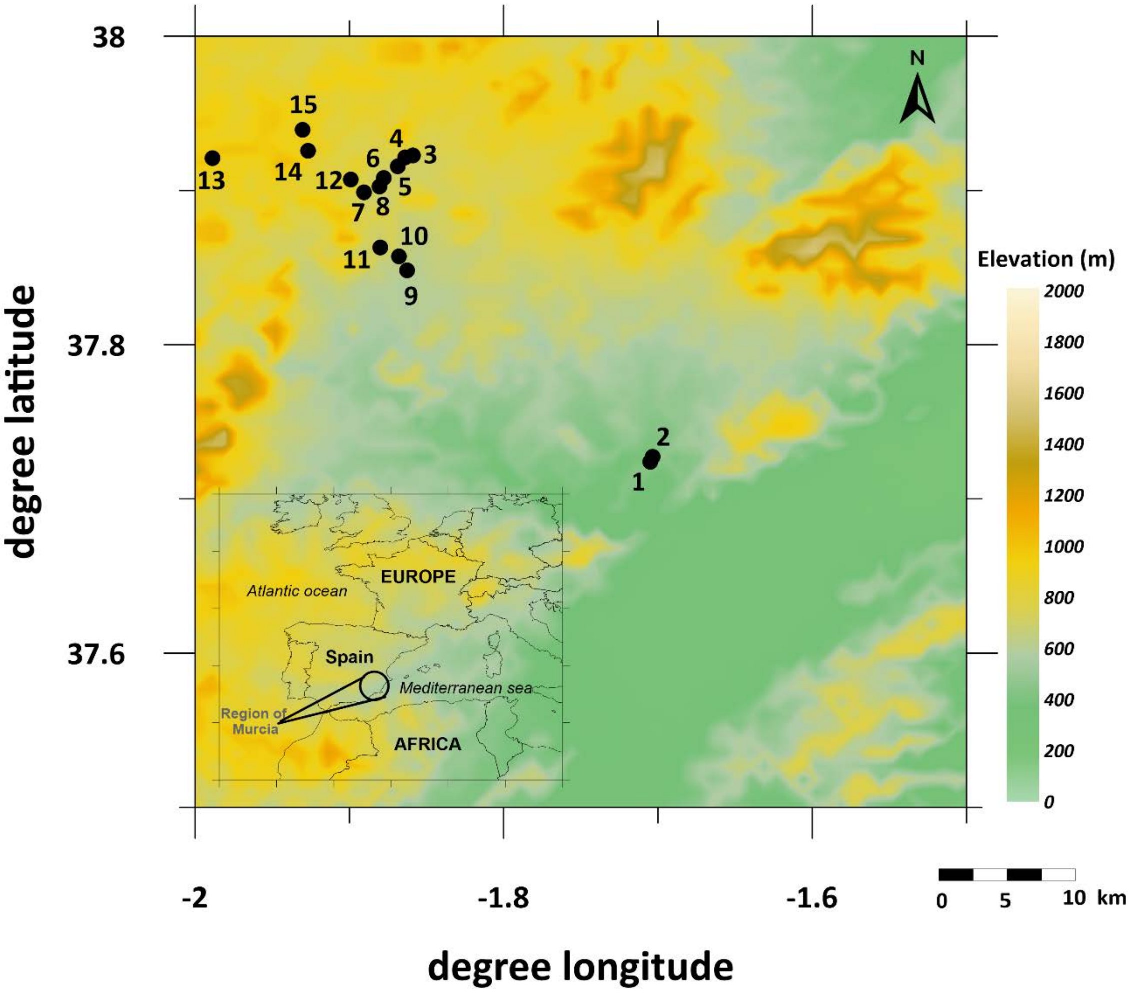

Supplement: Supplementary file 1 — Supplementary Information [file 42003_2019_700_MOESM1_ESM.pdf]
