# Supplementary material for: Phylogenetic rewiring in mycorrhizal–plant interaction networks increases community stability in naturally fragmented landscapes
Source: Commun Biol. 2019 Dec 5;2:452. doi: 10.1038/s42003-019-0700-3 (PMC6895200; doi:10.1038/s42003-019-0700-3)
Supplement: Supplementary file 2 — Descriptions of Additional Supplementary Files [file 42003_2019_700_MOESM2_ESM.docx]

Descriptions of additional supplementary files:

**Supplementary data 1.**

The data extends beyond the width of the page and thus is provided as separate files (.xlsx).

Supplementary data 1. Species composition in each fragment: Sheet (plants): Area, plant species composition, plant species abundance, and number of plant individuals sampled; Sheet (Fungal OTUs): presence (1) and absence (0) of the fungal operational taxonomic units in each fragment.

**Supplementary data 2.**

The data extends beyond the width of the page and thus is provided as separate files (.xlsx).

Supplementary data 2. Relative contributions of species turnover and rewiring to changes in the interaction patterns between pairs fragments. The interaction networks in the 15 fragments are considered at the species level. Each row represents the comparison of two networks obtained in different fragments. β_WN_ (dissimilarity of interactions), β_OS_ (dissimilarity of interactions due to rewiring), β_ST_ (dissimilarity of interactions due to species turnover), β_ST/WN_ (contribution of species turnover (relative to rewiring) to interactions dissimilarity) (*sensu* Poisot et al. 2012) (29).

**Supplementary data 3.**

The data extends beyond the width of the page and thus is provided as separate files (.xlsx).

Supplementary data 3. Mean estimates of robustness [95% confidence interval] in the plant-mycorrhizal fungi network in each fragment. Robustness is estimated under different scenarios: without rewiring (“noRW”) and allowing random (RWrand) and phylogenetically constrained rewiring (“RWphylo”). For each fragment, it is presented its area, number of realized and non-realized (despite the two partners are present) interactions, number of plant individuals, number of species (operational taxonomic units in the case of fungi), and number of families sampled for plants and fungi, respectively.

**Supplementary Code 1.**

The R code used to perform the simulations to assess whether the rewiring of mycorrhizae interactions is phylogenetically conserved is provided as a (.txt) file. http://dx.doi.org/10.20350/digitalCSIC/9064
