# Supplementary material for: Phylogenetic rewiring in mycorrhizal–plant interaction networks increases community stability in naturally fragmented landscapes
Source: Commun Biol. 2019 Dec 5;2:452. doi: 10.1038/s42003-019-0700-3 (PMC6895200; doi:10.1038/s42003-019-0700-3)
Supplement: Supplementary file 8 — Reporting Summary [file 42003_2019_700_MOESM8_ESM.pdf]

nature research

Corresponding author(s): Alicia Montesinos Navarro

Last updated by author(s): Nov 14, 2019

Reporting Summary

Nature Research wishes to improve the reproducibility of the work that we publish. This form provides structure for consistency and transparency in reporting. For further information on Nature Research policies, see Authors & Referees and the Editorial Policy Checklist.

Statistics

For all statistical analyses, confirm that the following items are present in the figure legend, table legend, main text, or Methods section.

n/a	Confirmed	
☐	☒	The exact sample size (*n*) for each experimental group/condition, given as a discrete number and unit of measurement
☒	☐	A statement on whether measurements were taken from distinct samples or whether the same sample was measured repeatedly
☐	☒	The statistical test(s) used AND whether they are one- or two-sided<br>*Only common tests should be described solely by name; describe more complex techniques in the Methods section.*
☐	☒	A description of all covariates tested
☒	☐	A description of any assumptions or corrections, such as tests of normality and adjustment for multiple comparisons
☐	☒	A full description of the statistical parameters including central tendency (e.g. means) or other basic estimates (e.g. regression coefficient) AND variation (e.g. standard deviation) or associated estimates of uncertainty (e.g. confidence intervals)
☐	☒	For null hypothesis testing, the test statistic (e.g. *F*, *t*, *r*) with confidence intervals, effect sizes, degrees of freedom and *P* value noted<br>*Give P values as exact values whenever suitable.*
☒	☐	For Bayesian analysis, information on the choice of priors and Markov chain Monte Carlo settings
☒	☐	For hierarchical and complex designs, identification of the appropriate level for tests and full reporting of outcomes
☒	☐	Estimates of effect sizes (e.g. Cohen's *d*, Pearson's *r*), indicating how they were calculated

Our web collection on statistics for biologists contains articles on many of the points above.

Software and code

Policy information about availability of computer code

Data collection	We did not use any code to collect data in this study
Data analysis	all analyses were performed in R software version 3.5.2. R Core Team, R: A language and environment for statistical computing. R Foundation for Statistical Computing, Vienna, Austria. 2013, (2015). R code is provided as supplementary material

For manuscripts utilizing custom algorithms or software that are central to the research but not yet described in published literature, software must be made available to editors/reviewers. We strongly encourage code deposition in a community repository (e.g. GitHub). See the Nature Research guidelines for submitting code & software for further information.

Data

Policy information about availability of data

All manuscripts must include a data availability statement. This statement should provide the following information, where applicable:
- Accession codes, unique identifiers, or web links for publicly available datasets
- A list of figures that have associated raw data
- A description of any restrictions on data availability

The data used is available in the National Center for Biotechnology Information Search database (NCBI); Sequence Read Archive (SRA) accession: PRJNA516318

Field-specific reporting

Please select the one below that is the best fit for your research. If you are not sure, read the appropriate sections before making your selection.

☐ Life sciences ☐ Behavioural & social sciences ☒ Ecological, evolutionary & environmental sciences

Ecological, evolutionary & environmental sciences study design

All studies must disclose on these points even when the disclosure is negative.

Study description	We analyzed shifts in mycorrhizal interactions across 15 lithological fragments (gypsum outcrops) with different areas (range 0.15-85 ha). Our study system was a naturally fragmented gypsum area located in Southeastern Spain (37º40´N, 1º41´W). The climate is semiarid Mediterranean, with a mean annual temperature of 16 ºC, average annual rainfall of 289 mm, mainly concentrated in early spring and fall, and a pronounced drought season in summer. Gypsum ecosystems are very restrictive environments which occur in arid and semiarid regions and are subject to particularly stressful conditions due to the properties of the soil and climate. These ecosystems usually have a fragmented spatial structure because the soil has a natural patchy distribution, as a mosaic of edaphic islands immersed within other lithologies or geological substrates.
Research sample	In order to have representative samples of the plant communities, we estimated the plant species composition and abundance in each fragment using line-transects, which were proportional in length to the area of the fragment. The number of individuals for each plant species was recorded and the relative abundance of each plant species was determined. Based on the relative abundances of the plant species in each fragment, 15 plant individuals were selected for sampling (Table S1).<br>Plant individuals were collected in May 2015 (late spring) along a line-transect in the core of each fragment, to avoid major edge effects. We sampled 15 individuals per fragment for a total of 225 individuals belonging to 28 plant species. Roots of each individual were genotyped using next geneation sequencing to characterize the arbuscular mycorrhizal community in each plant.
Sampling strategy	Plant individuals were hazarly selected to have a representative sample of the relative abundance of the different plant species present in each community. The costs of next generation sequencinng limited the analyses to a fixed numebr of samples in each of the 15 sites, in order to have comparable networks across sites.
Data collection	The samples were collected by the authors during May 2015, and the bioinformatic analyses were performed by All Genetics & Biology, SL, A Coruña, Spain.
Timing and spatial scale	All the samples are collected sequentially during the month of May 2015, at a regional scale in southestern Spain.
Data exclusions	No data were excluded from the analyses
Reproducibility	Next Generation Sequencing (NGS) approaches are used as a tool to study arbuscular mycorrhizal fungi communities as they provide sufficient depth to deliver insights into variations in environmental samples. These approaches allow infrequent fungi to be detected and increase the proportions of diversity captured (20). The Illumina sequencing platform is a particularly useful approach because of its low error rate and high sequencing depth per sample. We used the Illumina MiSeq platform 2 x 300 bp, which produces longer DNA reads than the previous 2 x 200 bp length platform, and an amplicon-based and tagmentation approach (i.e. universal tags are incorporated into DNA fragments) in order to allow longer DNA regions, such as the 18 s rDNA region, to be sequenced.<br>Total DNA was extracted from 500 mg of roots of each plant individual (sample) by using the MOBIO Power Soil® DNA Isolation kit (MO BIO Laboratories, Inc., Carlsbad, CA, USA). After the procedure, the concentrations of DNA in the samples were measured using an Appliskan fluorescence-based microplate reader (Thermo Scientific Wilmington, US). Amplicon library preparation for the Illumina MiSeq platform was based on the Illumina MiSeq system preparation manual “16S Metagenomic Sequencing Library Preparation”. The region-specific primers NS31 and AML2 (21, 22) were used to target the 18s rDNA V4 region of arbuscular mycorrhizal fungi, with overhang adapters attached.<br>Thus, the composite primers were forward 5’ TCGTCGGCAGCGTCAGATGTGTATAAGAGACAGTTGGAGGGCAAGTCTGGTGCC and reverse 5’ GTCTCGTGGGCTCGGAGATGTGTATAAGAGACAGGAACCCAAACACTTTGGTTTCC. For Index PCR, Illumina Nextera XT v2 Index Kit primers were used. The forward index primer was: AATGATACGGCGACCACCGAGATCTACAC[i5]TCGTCGGCAGCGTC; and the reverse index primer was: CAAGCAGAAGACGGCATACGAGAT[i7]GTCTCGTGGGCTCGG, where i5 and i7 indicate the position of forward and reverse index sequences.<br>The PCR was carried out in two sequential reactions: (1) Amplicon PCR with region-specific primers and partial adapters for sequencing; (2) Index PCR, which was used to complete the sequencing adapters and add Nextera XT sample identifying indexes. Between sequential reactions, the amplicons were purified with AMPure bead technology. In the Amplicon PCR 3 μL of DNA sample were used, while 3 μL of purified amplicon were used in the Index PCR reaction.<br>The total reaction volume was 30 μL. The PCR reactions contained primers (0.2 μM, final concentrations) and Smart-Taq Hot Red 2x PCR Mix (0.1 U/μL Smart Taq Hot Red Thermostable DNA Polymerase, 4 mM MgCl2, 0.4 mM dATP, 0.4 mM dCTP, 0.4 mM dGTP, 0.4 mM dTTP; Naxo OÜ, Estonia). An NTC (no template control) was added to all PCR reactions. A 1-kb DNA Ladder (Naxo OÜ, Estonia) was used as a size marker on all electrophoresis gels.<br>Amplicons of the 18s DNA V4 region produced by Index PCR were purified with an Agencourt AMPureXP Kit (Beckman Coulter Inc. Brea, US Samples were eluted in Buffer EB (10 mM Tris-HCl, pH 8.5; QIAGEN Inc). The DNA concentrations of the purified amplicons were measured using an Appliskan fluorescence-based microplate reader (Thermo Scientific) and PicoGreen® dsDNA Quantitation Reagent (Quant-iT dsDNA Broad Range Assay Kit, Invitrogen Wilmington, US After measurement of the concentrations of the amplicons, those with a concentration of at least 10 ng/μL were pooled equimolarly (200 ng of each sample). The concentration of DNA in the pool was measured in a QubitTM fluorometer, in three replicates, resulting in a mean value of 43.9 ng/μL. Twelve samples failed to yield a detectable amplicon in the Index PCR and thus were excluded from the sequencing library.<br>The DNA extraction and the preparatory procedures for sequencing were performed using BIOTAP LLC (Tallinn, Estonia). The sequencing of the DNA library was performed on the Illumina MiSeq 2 x 300 bp v3 run platform by Microsynth AG (Balgach, Switzerland).<br>Bioinformatics<br>Demultiplexing and Illumina adaptor trimming were performed, and Row R1 and R2 Miseq reads were obtained in FASTQ format. The quality of the obtained reads was checked with the software FASTQC (http://www.bioinformatics.babraham.ac.uk/projects/fastqc/). The processing of the FASTQ files, containing the reads, was carried out with Qiime 1.9.0 (23), which performs standard microbial

	community analyses, including quality filtering of reads, operational taxonomic unit (OTU) picking, and taxonomic assignment. After quality filtering of the reads (minimum Phred quality score of 20), chimeras were detected and removed using the UCHIME algorithm implemented in VSEARCH (24). The remaining filtered and cleaned 18S reads were then clustered into OTUs through the closed-reference approach in Qiime, using the MaarjAM database as the reference (25). Each OTU was assigned to fungal taxa using the UCLUST algorithm (26). Data on the OTU abundance in each sample at different taxonomic levels were obtained. Then, a second quality filtering was carried out: we removed the OTUs with a number of sequences lower than 0.005 % of the total number of sequences. Finally, the rarefaction plots were constructed, to show the rarefied number of OTUs defined at a 97 % sequence similarity threshold. When the rarefaction curves tended towards saturation, the sequencing depth was assumed to be sufficient to retrieve most of the arbuscular mycorrhizal diversity. In addition, the percentage of coverage was calculated by Good's method (27).
Randomization	Groups os samples were difined by the different 15 locations. In each location 15 individuals of the mos abundant speices were sampled based on their relative abundance.
Blinding	Mycorrhizal community was charachterized based on next generation sequencing, and therefore the community of fungi in each plant can be considered blind to the sapling protocol

Did the study involve field work? ☒ Yes ☐ No

Field work, collection and transport

Field conditions	The climate is semiarid Mediterranean, with a mean annual temperature of 16 ºC, average annual rainfall of 289 mm, mainly concentrated in early spring and fall, and a pronounced drought season in summer. Gypsum ecosystems are very restrictive environments which occur in arid and semiarid regions and are subject to particularly stressful conditions due to the properties of the soil and climate. These ecosystems usually have a fragmented spatial structure because the soil has a natural patchy distribution, as a mosaic of edaphic islands immersed within other lithologies or geological substrates. The soils are classified as Petrogypsic Gypsiorthid (SSS, 1999) and Gypsisol (FAO, 1998), with a gypsic and petrogypsic horizon within 100 m of the surface developed on gypsum parental rocks. The vegetation is an open (40% vegetation coverage) scrubland with woody gypsophilous perennial species, and other plant species that do not exclusively grow in gypsum soils. The plant community is rich in germanders (Teucrium), thymes (Thymus), rockroses (Helianthemum), ruptureworts (Herniaria), composites, and grasses.
Location	Our study system was a naturally fragmented gypsum area located in Southeastern Spain (37º40´N, 1º41´W).
Access and import/export	We access the study sites using regular roads, and we did not need importation permits as all the samples were processed within the same country.
Disturbance	This study did not imply any important disturbance in the natural habitats

Reporting for specific materials, systems and methods

We require information from authors about some types of materials, experimental systems and methods used in many studies. Here, indicate whether each material, system or method listed is relevant to your study. If you are not sure if a list item applies to your research, read the appropriate section before selecting a response.

Materials & experimental systems

n/a	Involved in the study	
☒	☐	Antibodies
☒	☐	Eukaryotic cell lines
☒	☐	Palaeontology
☒	☐	Animals and other organisms
☒	☐	Human research participants
☒	☐	Clinical data

Methods

n/a	Involved in the study	
☒	☐	ChIP-seq
☒	☐	Flow cytometry
☒	☐	MRI-based neuroimaging
